# Association of Magnesium Intake with Liver Fibrosis among Adults in the United States

**DOI:** 10.3390/nu13010142

**Published:** 2021-01-02

**Authors:** Meng-Hua Tao, Kimberly G. Fulda

**Affiliations:** 1 Department of Biostatistics and Epidemiology, University of North Texas Health Science Center, Fort Worth, TX 76107, USA; 2 Department of Family Medicine and Osteopathic Manipulative Medicine, NorTex, University of North Texas Health Science Center, Fort Worth, TX 76107, USA; kimberly.fulda@unthsc.edu

**Keywords:** magnesium, calcium, significant liver fibrosis, epidemiology

## Abstract

Liver fibrosis represents the consequences of chronic liver injury. Individuals with alcoholic or nonalcoholic liver diseases are at high risk of magnesium deficiency. This study aimed to evaluate the association between magnesium and calcium intakes and significant liver fibrosis, and whether the associations differ by alcohol drinking status. Based on the National Health and Nutrition Examination Survey (NHANES) 2017–2018, the study included 4166 participants aged >18 years who completed the transient elastography examination and had data available on magnesium intake. The median liver stiffness of 8.2 kPa was used to identify subjects with significant fibrosis (≥F2). The age-adjusted prevalence of significant fibrosis was 12.81%. Overall total magnesium intake was marginally associated with reduced odds of significant fibrosis (*p* trend = 0.14). The inverse association of total magnesium intake with significant fibrosis was primarily presented among those who had daily calcium intake <1200 mg. There were no clear associations for significant fibrosis with calcium intake. Findings suggest that high total magnesium alone may reduce risk of significant fibrosis. Further studies are needed to confirm these findings.

## 1. Introduction

Chronic liver disease is a substantial worldwide problem [1]. Its major consequence is accumulation of extracellular matrix within the liver, leading to the development of cirrhosis, liver failure, or even liver cancer [2]. Nonalcoholic steatohepatitis (NASH), chronic hepatitis C infection, and alcohol abuse are the main causes of liver fibrosis in Western countries [2]. NASH is a subtype of nonalcoholic fatty liver disease (NAFLD) [3]. As the most common liver disease in the world [4], NAFLD is considered as the hepatic manifestation of metabolic syndrome [3] and is associated with obesity and type 2 diabetes [5]. Previous studies have also shown that liver fibrosis stage is associated with long-term outcomes of patients with NAFLD [6]. Inflammation, oxidant stress, and insulin resistance not only play critical roles in the progression of hepatic fibrosis in NAFLD patients [2], but they can occur and stimulate liver fibrosis among patients with hepatitis C or B infection [2,7] or alcoholic liver diseases [2,8].

Magnesium status is closely linked with liver function and may be related to the etiology of chronic liver disease. In the liver, mast cells contribute to liver fibrosis [9]; animal studies have shown that low-magnesium diet increases the levels of mRNA known to be expressed by mast cells in the liver and induce the emergence of mast cells around portal triads of the liver in Sprague–Dawley rats [9]. An in vivo study showed that extracellular magnesium deficiency modulates the expression levels of molecules related to oxidative stress/antioxidant response in HepG2 human hepatoma cells [10]. Serum magnesium concentration is also significantly low in patients with alcoholic steatosis, nonalcoholic steatosis, or NASH [11,12]. Previous studies found that chronic alcohol consumption leads to a decrease in liver magnesium content, while deficient magnesium levels in alcoholic liver disease patients, in turn, disrupt normal metabolism and lead to greater lipid deposition in the liver [13]. Recent studies suggest that high magnesium intake may be associated with reduced risk of fatty liver disease [14] and mortality due to liver disease, particularly among those with fatty liver disease or alcoholic drinkers [15]. Animal studies have shown that magnesium deficiency induces inflammatory response [16]; randomized controlled trials further report that magnesium treatments significantly decrease concentrations of C-reactive protein (CRP) among patients with metabolic syndrome [17] or high risk of inflammation [18]. Randomized controlled trials also show that magnesium supplementation improves insulin sensitivity in patients with type 2 diabetes or non-diabetic individuals with insulin resistance [19,20,21].

In this study, we investigated whether magnesium intake is associated with the prevalence of liver fibrosis in adults. Previous studies have examined the association of calcium intake with type 2 diabetes [22]; however, few studies have investigated the role of calcium intake in relation to liver fibrosis [23]. Calcium intake may interact with intake of magnesium in the development of many chronic diseases including fatty liver disease and prediabetes [14]. Therefore, we hypothesized that intake of calcium may also be associated with risk of liver fibrosis. To test this hypothesis, we examined the association between calcium intake and liver fibrosis and investigated whether the associations varied by alcohol drinking status among US adults utilizing data from the National Health and Nutrition Examination Survey (NHANES) conducted in 2017 and 2018.

## 2. Materials and Methods

### 2.1. Study Population

This study utilized data from one cycle of the National Health and Nutrition Examination Survey (NHANES) conducted between 2017 and 2018 in which liver ultrasound transient elastography examination was performed. The NHANES is an ongoing survey program designed to assess health and nutrition in a nationally representative sample of the non-institutionalized US population. A detailed description of the study design has been published elsewhere [24]. The survey is maintained and administered by the National Center for Health Statistics (NCHS) of the Centers for Disease Control and Prevention (CDC) [24]. In 2017–2018, 9254 persons completed the interview. In our study, participants who were aged less than 19 years at time of the survey and did not complete the liver ultrasound transient elastography examination were excluded (N = 4262). Pregnant or lactating females, participants with unreliable dietary data or autoimmune liver disease, and participants with missing data for magnesium, calcium intake, or confounders (age, sex, race/ethnicity, education, body mass index (BMI), high-density lipoprotein (HDL) cholesterol) were further excluded from the analyses (N = 826). As a result, 4166 participants were included in the final analysis. All participants provided written informed consent and the NCHS Research Ethics Review Board approved the survey protocol (Protocol #2011-17, 2018-01).

### 2.2. Ascertainment of Outcomes 

In the 2017–2018 cycle of NHANES, transient elastography measurements were conducted in the NHANES Mobile Examination Center (MEC) by trained health technicians, using FibroScan® model 502 V2 Touch equipped with a medium or extra-large probe. Briefly, transient elastography is a widely used and validated technique to quantitatively assess tissue stiffness. It is considered as a reliable, non-invasive method to assess liver fibrosis [25,26]. All participants aged 12 years and over were eligible with exclusion for participants who were unable to lie down on the exam table, pregnant at the time of their exam, had an implanted electronic medical device, or were wearing a bandage or had lesions on the site where measurements would be taken. Only participant with complete exams (i.e., fasting time of at least 3 h, 10 or more complete stiffness measures, and a liver stiffness interquartile range/median <30%) were included in the current analysis. Several meta-analyses [25,27] and a recent prospective study [28] analyzed, assessed, and reported optimal stiffness cutoff values to define different stages of fibrosis among subjects with NAFLD. The transient elastography cutoff values of 8.2, 9.7, and 13.6 (Kpa) were used to define METAVIR (Meta-analysis of Histological Data in Viral Hepatitis) fibrosis stage F2, F3, and F4 fibrosis, respectively [25,28]. A median liver stiffness of 8.2 (kPa) was used to define cases of significant fibrosis (≥F2).

### 2.3. Assessments of Nutrient Intake

Details of the protocol and dietary information collection methods have been described previously [29]. Briefly, daily dietary intake information was obtained through 24-h recall interviews and a 30-day dietary supplement questionnaire. Two 24-h recalls were conducted for each participant in NHANES 2017–2018. The first dietary recall was collected in person by trained interviewers in the NHANES MEC and the second dietary recall was completed by trained interviewers via telephone 3–10 days after the MEC interview [29]. To keep intake information consistent, only the in-person dietary recall for all participants was used in the current analysis. Total intakes of magnesium, calcium, and other nutrients were calculated by summing intake from foods and dietary supplements.

### 2.4. Assessments of Covariates

In the NHANES, race/ethnicity was categorized by using survey questions on race and Hispanic origin: non-Hispanic White referring to whites who are not of Hispanic origin; non-Hispanic Black referring to blacks without Hispanic origin; Hispanic referring to all Hispanics regardless of race; non-Hispanic Asian including Asians without Hispanic origin; and Other Race including American Indians or Alaska Natives, Native Hawaiians or other Pacific Islanders, and multiracial persons. The amount of daily alcohol beverage consumption was also collected in the 24-h recalls. Daily alcohol consumption was categorized into non-drinkers (0 g), low (<31.32 g), and high (≥31.32 g) based upon the median daily alcohol intake among non-cases. Body mass index (BMI, measured weight/height^2^) was classified into three categories: <25.0, 25.0–29.9, and ≥30.0, categories of under/normal weight, overweight, and obesity, respectively. Based on Physical Activity Guidelines recommendation of at least 75 min of vigorous or 150 min of moderate physical activity in a typical week [30], participants were classified into physically inactive (no), less active (<the recommendation), and active (≥the recommendation). Participants with type 2 diabetes were identified as having any of the following: (1) hemoglobin A1C concentration ≥6.5% [31] or (2) self-report of diabetes diagnosis. Participants with hepatitis B or C virus (HBV or HCV) infection were identified by positive testing results [32,33] or self-report of hepatitis B or C infection. Participants were asked whether they had ever smoked ≥100 cigarettes in their lifetime and whether they smoked currently to identify current and former smokers. Participants were defined as former smokers if they did not smoke currently but had ever smoked ≥100 cigarettes in the past. Blood high-density lipoprotein (HDL) cholesterol was measured based on standard laboratory methods.

### 2.5. Statistical Analysis

All statistical analyses were conducted in SAS 9.4 software (SAS Institute, Cary, NC, USA) using the “Survey” procedure to estimate variance after incorporating the complex, multistage, clustered probability sampling design of the NHANES [34]. Characteristics and covariates were compared between those with and without significant fibrosis using Rao–Scott chi-square test for categorical variables and Student’s *t*-test for continuous variables. The age-adjusted prevalence of significant fibrosis was estimated, stratified by race/ethnicity and age groups using the 2000 US Census as the standard population. Logistic regressions were used to estimate odds ratios (ORs) and 95% confidence intervals (95% CIs) for associations between magnesium intake and significant fibrosis. Dietary and total magnesium and calcium intake were categorized into quartiles based on intakes among non-cases, using the lowest category as the reference. Potential confounders included in analyses were gender, race/ethnicity, education, BMI status, alcohol intake status, physical activity status, HCV infection status, and medical history of diabetes. Age and HDL, as well as daily intakes of total energy, calcium, magnesium, fiber, and phosphate, were included as continuous variables for adjustment in models. We also assessed potential confounding by smoking and HBV status, and these factors did not alter point estimates by ≥10% and were excluded from the final models. Tests for dose–response relationship were estimated by fitting models with exposure variables included as continuous variables.

Stratified analyses by gender, calcium:magnesium intake ratio (<2.62, ≥2.62, according to physiological range of the ratio and previous reports on the ratio in the US population [14,35]; the second quartile of the ratio in our population was used as the cut-point), daily calcium intake (<1200 mg or ≥1200 mg), daily alcohol drinking status (no, yes), and race/ethnicity were further conducted. Possible interaction between magnesium or calcium intake and potential effect modifiers were examined in the logistic regression model by evaluation of multiplicative interactions. All reported *p*-values are two-sided with statistical significance evaluated at 0.05.

## 3. Results

Table 1 summarizes selected characteristics by significant liver fibrosis status. A total of 628 participants presented ≥F2 liver fibrosis, with age-adjusted prevalence of 12.81%. Compared to participants without significant fibrosis, those with ≥F2 fibrosis were older, more likely to be male, obese, and physically inactive, and had a history of HCV infection or diabetes, lower HDL level, and higher total energy intake.

Associations of intakes of magnesium and calcium with the odds of significant fibrosis are presented in Table 2. After adjustment for intakes of energy, fiber, phosphates and total calcium, age, race/ethnicity, gender, and other potential confounders, total magnesium intake was marginally associated with lower odds of significant fibrosis (OR = 0.53, 95% CI, 0.35–1.10; *p* trend = 0.14). When examining dietary magnesium intake, no significant association was observed. Neither dietary nor total intake of calcium was associated with odds of significant fibrosis.

We further conducted stratified analyses for total magnesium intake by the ratio of calcium:magnesium intake (Table 3). Among those with a high calcium:magnesium intake ratio (≥2.62), participants who consumed high total magnesium tended to have lower odds of significant fibrosis; however, the association was not statistically significant. Among those with a low calcium:magnesium intake ratio (<2.62), the pattern suggested a positive association between high magnesium intake and odds of significant fibrosis. Again, the association was not statistically significant, possibly due to a smaller sample size in this strata. In addition, there was no significant interaction between calcium:magnesium ratio and intake of magnesium. On the other hand, among those who had a daily total calcium intake <1200 mg, compared to those with the lowest quartile intake, participants with total magnesium intake at the highest quartile had reduced odds of significant fibrosis (OR = 0.35, 95% CI, 0.16–0.77; *p* trend = 0.02). The test for interaction between magnesium intake and calcium intake was not significant (*p* interaction = 0.44). No significant interactions were found between magnesium intake and other potential effect modifiers including daily alcohol drinking status (*p* interaction = 0.38) and gender (*p* interaction = 0.75). However, the reduced odds of significant fibrosis in relation to high total magnesium intake tended to be stronger in non-drinkers (OR = 0.45, 95% CI, 0.18–1.09; *p* trend = 0.09) and males (OR = 0.47, 95% CI, 0.23–0.99; *p* trend = 0.12). Similarly, we found the inverse association of total magnesium intake with odds of significant fibrosis only among non-Hispanic whites (OR = 0.34, 95% CI, 0.12–0.98 the highest vs. the lowest quartile; *p* trend = 0.10) (data not shown). No clear associations were observed among other racial/ethnic groups, possibly due to smaller sample sizes for these racial/ethnic groups. Again, no significant interaction was found for total magnesium intake and race/ethnicity.

Intake of total calcium was not significantly associated with significant fibrosis in both males and females (Table 4). No significant association or interaction was found for total calcium intake in different groups of calcium:magnesium intake or alcohol drinking status. The association between intake of calcium and significant fibrosis did not differ by race/ethnicity.

## 4. Discussion

Utilizing data from the recent NHANES cycle, a nationally representative sample of the US general population, results suggested that there is an association between high total magnesium intake and lower odds of significant liver fibrosis. Moreover, the inverse association between magnesium intake and significant fibrosis appeared stronger among males, non-Hispanic whites, subjects who had calcium intake <1200 mg per day, and subjects who did not drink alcohol, although interactions were not statistically significant. On the other hand, there was no association between calcium intake and significant fibrosis.

Our finding of the inverse association between total magnesium intake and significant fibrosis is consistent with previous studies. Rodríguez-Hernández et al. [36] reported a positive association between low serum magnesium and risk of NASH in a study of obese subjects. A recent study using data from NHANES III found a significant association of total magnesium intake with reduced odds of fatty liver disease [14]. In particular, our results suggest an inverse association between total magnesium intake and odds of significant fibrosis among subjects who did not drink alcoholic beverages. Our novel findings are consistent with previous results that serum magnesium concentrations were significantly lower in individuals with NASH [12,36]. This finding is important because the prevalence of NAFLD has been increasing steadily in the last three decades in the US with an estimated prevalence of 32% in adults [37]. Meanwhile, there are steady increases in the rates of obesity and diabetes in the US [37], both of which are major risk factors of NAFLD [5]. It has been reported that 60% of American adults do not meet the Estimated Average Requirement (EAR) for magnesium in NHANES 2001–2008 [38], and overweight/obese adults had higher prevalence of inadequate intake of magnesium than normal-weight adults [38,39]. Further large studies are needed to confirm the results.

Previous studies have shown the importance of the balance between magnesium and calcium in relation to their physiological functions. Calcium can directly or indirectly compete with magnesium for (re)absorption in the intestine and kidney [40]. Clinical trials consistently show that a high calcium and low or insufficient magnesium diet can interfere with magnesium absorption, resulting in depressed absorption of magnesium [40,41]. In agreement with prior studies on fatty liver disease [14], our study found that total magnesium intake was associated with reduced odds of significant liver fibrosis only among those with daily calcium intake less than 1200 mg, suggesting that the beneficial effects of magnesium could be suppressed when calcium intake is more than the Dietary Reference Intakes (DRIs).

The inverse association between total magnesium intake and significant fibrosis was limited among non-Hispanic whites in our study. Previous studies found that non-Hispanic whites had higher magnesium intake than non-Hispanic blacks and Hispanics [39], which may be in part due to disparities in socioeconomic status and educational attainment between racial/ethnic groups. The relatively small number of minority participants still limited our ability to detect weak associations in each minority group. Further studies are needed to understand specific associations in minority populations.

The strengths of this study include using NHANES data with nationally representative samples and a relatively large number of adults with transient elastography examination, providing the power to detect weak associations. However, several limitations common to observational studies should be mentioned. Due to the nature of cross-sectional studies, the temporal sequences may not be clear. However, the suggested inverse association between magnesium intake and significant fibrosis is consistent with previous findings on the associations of magnesium intake with fatty liver disease [14], metabolic syndrome, and insulin resistance [17,20,21]. Although the transient elastography measurement is a widely used non-invasive method to assess liver fibrosis [25,26], it can be limited by patient obesity, the presence of perihepatic ascites, and limited selection of an appropriate sampling area [42]. However, the transient elastography has been shown as a validated and reliable technique and has been recommended to discriminate significant (F ≥ 2) from non-significant fibrosis (F0–F1) by the World Federation for Ultrasound in Medicine and Biology [42]. In addition, misclassification may have occurred in the analyses since there is no well-defined cutoff for significant fibrosis utilizing the transient elastography measurement. However, this misclassification is likely to be nondifferential. Alcohol intake is an important risk factor for liver fibrosis; we adjusted for the amount of alcohol intake based on the 24-h dietary recall due to the unavailability of data from the alcohol use questionnaire (ALQ) in the NHANES 2017–18 cycle. Previous studies showed that the frequency of participants consuming some amount of alcoholic beverages estimated through the 24-h dietary recall was lower than the frequency of drinking some alcoholic beverages at least once in the past one-year period obtained from the ALQ [43]. The residual confounding by alcohol intake in this study may dilute or even mask the associations of magnesium intake with liver fibrosis. The 24-h dietary recall used in NHANES has been extensively evaluated [29]; however, the self-reported dietary recall is likely to have both random and systematic errors, particularly in energy intake [44]. Moreover, recall bias from self-reported diet may also exist [45]. Although multiple 24-h dietary recalls are used as the gold standard measure in large-scale nutrition epidemiological studies, a one-time, 24-h dietary recall may not capture long-term dietary exposures [29]. We adjusted for many potential confounding factors including physical activity, daily alcohol drinking status, and several medical conditions associated with liver fibrosis, which enabled us to capture the association more accurately.

In conclusion, our findings suggest that participants who had high intake of magnesium may have reduced odds of having significant liver fibrosis, whereas high intake of calcium was not associated with change in risk. In particular, the inverse association may be limited among those who had daily calcium intake less than 1200 mg and those who did not drink alcohol. Further studies, such as prospective cohort studies, are warranted to confirm the present results.

## Figures and Tables

**Table 1:** Participant characteristics by significant liver fibrosis status, National Health and Nutrition Examination Survey (NHANES) 2017–2018.

Characters	Yes (*n* = 628)	No (*n* = 3538)	*p*-Value
Age (years) ^ǂ^	52.4 (1.36)	47.7 (0.68)	<0.01
Sex, n (%)*			<0.01
Male	371 (58.8)	1698 (48.1)	
Female	257 (41.2)	1840 (51.9)	
Race/ethnicity, n (%)*			0.44
Non-Hispanic White	230 (65.3)	1501 (64.5)	
Non-Hispanic Black	161 (11.8)	950 (10.2)	
Hispanic	158 (15.4)	956 (15.1)	
Non-Hispanic Asian	53 (3.4)	549 (5.4)	
Other races ^1^	26 (4.1)	210 (4.8)	
Education, n (%)*			0.03
Less than high school	131 (11.2)	637 (10.0)	
High school	156 (31.9)	852 (27.3)	
Some college	229 (35.4)	1147 (30.2)	
College graduate	112 (21.5)	902 (32.5)	
BMI group, n (%)*			<0.01
<25	89 (13.3)	975 (28.4)	
25–30	130 (18.0)	1200 (32.8)	
≥30	409 (68.7)	1363 (38.8)	
Physical activity level, n (%)*			<0.01
Active	142 (22.8)	961 (30.5)	
Less active	107 (22.2)	792 (26.5)	
Inactive	379 (54.9)	1785 (43.0)	
Smoking status, n (%)*			0.14
Never	327 (51.7)	2050 (57.9)	
Former	186 (30.8)	842 (24.9)	
Current	115 (17.4)	646 (17.2)	
Daily alcohol drinking status, n (%)			0.73
Non-drinkers	499 (73.2)	2785 (74.2)	
Low drinkers (<31.32 g)	63 (12.1)	399 (13.2)	
High drinkers (≥31.32 g)	66 (14.7)	354 (12.6)	
History of diabetes, n (%)			<0.01
Yes	239 (32.3)	622 (11.8)	
Having HBV infection, n (%)			0.09
Yes	43 (3.9)	231 (3.8)	
Having HCV infection, n (%)			<0.01
Yes	48 (7.7)	64 (1.9)	
Laboratory features ^ǂ^			
HDL (mmol/L)	1.3 (0.03)	1.4 (0.01)	<0.01
Median CAP (dB/m)	306.8 (4.36)	258.9 (2.07)	<0.01
Median stiffness (kPa)	13.8 (0.54)	4.7 (0.04)	<0.01
Daily intake of nutrients ^ǂ^			
Total energy (kcal)	2325.6 (45.30)	2176.3 (18.39)	0.01
Dietary calcium (mg)	1005.9 (36.95)	968.5 (14.34)	0.33
Dietary magnesium (mg)	310.7 (11.8)	304.9 (4.28)	0.57
Total calcium (mg)	1101.1 (37.81)	1061.4 (17.27)	0.33
Total magnesium (mg)	329.3 (16.91)	329.8 (6.00)	0.97

^ǂ^ Values shown are mean and (standard error); * unweighted frequency counts and weighted percentages shown. ^1^ Other races include American Indian or Alaska Native, Native Hawaiian or other Pacific Islander, and multiracial persons.

**Table 2:** Associations between intakes of magnesium and calcium and odds of significant liver fibrosis, NHANES 2017–2018.

Daily Intake of Nutrients (mg)	Liver Fibrosis Status		Model 1	Model 2
	Yes	No	OR (95% CI) ^1^	OR (95% CI) ^2^
Dietary magnesium				
Q_1_ < 205.93	190	996	Referent	Referent
Q_2_ 205.93–279.12	154	866	0.74 (0.51–1.06)	0.73 (0.50–1.06)
Q_3_ 279.13–375.20	143	857	0.96 (0.60–1.54)	0.87 (0.49–1.54)
Q_4_ ≥ 375.21	141	819	0.99 (0.67–1.45)	0.70 (0.35–1.38)
*p*_trend_			0.77	0.47
Total magnesium intake				
Q_1_ < 212.99	189	1009	1.00	1.00
Q_2_ 212.99–294.38	162	861	0.70 (0.52–0.95)	0.70 (0.51–0.97)
Q_3_ 294.39–400.26	134	853	0.91 (0.56–1.47)	0.70 (0.38–1.28)
Q_4_ ≥400.27	143	815	0.85 (0.54–1.34)	0.53 (0.25–1.10)
*p*_trend_			0.74	0.14
Dietary calcium				
Q_1_ < 574.06	183	1047	Referent	Referent
Q_2_ 574.06–855.22	153	893	1.41 (0.86–2.32)	1.54 (0.97–2.45)
Q_3_ 855.23–1238.68	160	822	1.22 (0.84–1.77)	1.14 (0.77–1.67)
Q_4_ ≥1238.69	132	776	1.29 (0.85–1.96)	1.03 (0.59–1.79)
*p*_trend_			0.31	0.77
Total calcium intake				
Q_1_ < 628.54	177	1051	1.00	1.00
Q_2_ 628.54–945.25	170	883	1.41 (0.86–2.32)	1.50 (0.97–2.33)
Q_3_ 945.25–1356.52	146	823	1.19 (0.76–1.84)	1.10 (0.72–1.68)
Q_4_ ≥ 1356.53	135	781	1.23 (0.78–1.94)	1.14 (0.71–1.84)
*p*_trend_			0.53	0.99

^1^ Adjusted for age; ^2^ further adjusted for gender, race/ethnicity, education, BMI, physical activity status, HCV infection status, status of diabetes, HDL level, and intakes of total energy, alcohol, fiber, and phosphate. Intakes of calcium and magnesium are mutually adjusted.

**Table 3:** Associations between intake of total magnesium and odds of significant fibrosis by gender, calcium:magnesium ratio, intake of calcium, and daily alcohol drinking status, NHANES 2017–2018.

Total Magnesium Intake (mg/Day)	Significant Liver Fibrosis			
	Yes	No	OR (95% CI) ^1^	*p* for Trend
Males				
Q_1_ < 212.99	97	357	1.00	
Q_2_ 212.99–294.38	88	368	0.58 (0.36–0.95)	
Q_3_ 294.39–400.26	84	449	0.67 (0.32–1.43)	
Q_4_ ≥ 400.27	102	524	0.47 (0.23–0.99)	0.12
Females				
Q_1_ < 212.99	92	652	1.00	
Q_2_ 212.99–294.38	74	493	0.77 (0.52–1.15)	
Q_3_ 294.39–400.26	50	404	0.65 (0.35–1.20)	
Q_4_ ≥ 400.27	41	291	0.63 (0.18–2.19)	0.36
*p* for interaction				0.75
Calcium:Magnesium ratio < 2.62				
Q_1_ < 212.99	46	320	1.00	
Q_2_ 212.99–294.38	61	274	1.83 (1.05–3.21)	
Q_3_ 294.39–400.26	56	333	1.33 (0.53–3.31)	
Q_4_ ≥ 400.27	70	436	0.70 (0.26–1.91)	0.44
Calcium:Magnesium ratio ≥ 2.62				
Q_1_ < 212.99	143	689	1.00	
Q_2_ 212.99–294.38	101	587	0.52 (0.35–0.78)	
Q_3_ 294.39–400.26	78	520	0.59 (0.29–1.21)	
Q_4_ ≥ 400.27	73	379	0.59 (0.23–1.49)	0.29
*p* for interaction				0.30
Calcium < 1200 mg/day				
Q_1_ < 212.99	171	941	1.00	
Q_2_ 212.99–294.38	128	681	0.66 (0.46–0.96)	
Q_3_ 294.39–400.26	92	555	0.56 (0.29–1.10)	
Q_4_ ≥ 400.27	53	329	0.35 (0.16–0.77)	0.02
Calcium ≥ 1200 mg/day				
Q_1_ <212.99	18	68	1.00	
Q_2_ 212.99–294.38	34	180	0.71 (0.20–1.06)	
Q_3_ 294.39–400.26	42	298	0.53 (0.20–1.42)	
Q_4_ ≥400.27	90	486	0.38 (0.16–1.43)	0.43
*p* for interaction				0.44
Daily alcohol drinking: No				
Q_1_ < 212.99	161	858	1.00	
Q_2_ 212.99–294.38	134	683	0.72 (0.49–1.06)	
Q_3_ 294.39–400.26	106	650	0.69 (0.39–1.24)	
Q_4_ ≥ 400.27	98	594	0.45 (0.18–1.09)	0.09
Daily alcohol drinking: Yes				
Q_1_ < 212.99	28	151	1.00	
Q_2_ 212.99–294.38	28	178	0.60 (0.33–1.09)	
Q_3_ 294.39–400.26	28	203	0.57 (0.23–1.40)	
Q_4_ ≥ 400.27	45	221	0.68 (0.30–1.51)	0.51
*p* for interaction				0.51

^1^ Adjusted for age, gender, race/ethnicity, education, BMI status, physical activity status, alcohol intake status, HCV infection status, HBV infection status, status of diabetes, HDL level, and intakes of total energy, fiber, phosphate, and total calcium.

**Table 4:** Associations between intake of total calcium and odds of significant fibrosis by gender, calcium:magnesium ratio, and daily alcohol drinking status, NHANES 2017–2018.

Total Calcium Intake (mg/Day)	Significant Liver Fibrosis			
	Yes	No	OR (95% CI) ^1^	*p* for Trend
Males				
Q_1_ < 628.54	95	450	1.00	
Q_2_ 628.54–945.25	95	414	1.56 (0.96–2.54)	
Q_3_ 945.25–1356.52	91	393	1.07 (0.70–1.63)	
Q_4_ ≥ 1356.53	90	441	1.10 (0.58–2.07)	0.77
Females				
Q_1_ < 628.54	82	601	1.00	
Q_2_ 628.54–945.25	75	469	1.51 (0.77–3.00)	
Q_3_ 945.25–1356.52	55	430	1.23 (0.57–2.63)	
Q_4_ ≥ 1356.53	45	340	1.37 (0.50–3.75)	0.64
*p* for interaction				0.99
Calcium:Magnesium ratio < 2.62				
Q_1_ < 628.54	125	737	1.00	
Q_2_ 628.54–945.25	64	370	1.89 (0.93–3.83)	
Q_3_ 945.25–1356.52	32	181	2.40 (1.18–4.85)	
Q_4_ ≥ 1356.53	12	75	1.72 (0.32–9.20)	0.12
Calcium:Magnesium ratio ≥ 2.62				
Q_1_ < 628.54	52	314	1.00	
Q_2_ 628.54–945.25	106	513	1.99 (0.69–2.09)	
Q_3_ 945.25–1356.52	114	642	0.82 (0.47–1.42)	
Q_4_ ≥ 1356.53	123	706	0.91 (0.46–1.81)	0.45
*p* for interaction				0.17
Daily alcohol drinking: No				
Q_1_ < 628.54	151	837	1.00	
Q_2_ 628.54–945.25	139	695	1.29 (0.85–1.96)	
Q_3_ 945.25–1356.52	104	636	0.99 (0.69–1.42)	
Q_4_ ≥ 1356.53	105	617	0.92 (0.53–1.61)	0.51
Daily alcohol drinking: Yes				
Q_1_ < 628.54	26	214	1.00	
Q_2_ 628.54–945.25	31	188	2.17 (0.66–7.12)	
Q_3_ 945.25–1356.52	42	187	1.50 (0.37–6.11)	
Q_4_ ≥ 1356.53	30	164	2.40 (0.37–15.77)	0.51
*p* for interaction				0.59

^1^ Adjusted for age, gender, race/ethnicity, education, BMI status, physical activity status, alcohol intake status, HCV infection status, HBV infection status, status of diabetes, HDL level, and intakes of total energy, fiber, phosphate, and total magnesium.

## Data Availability

Publicly available datasets were analyzed in this study. This data can be found here: https://wwwn.cdc.gov/nchs/nhanes/.

## References

[1] Asrani S.K., Devarbhavi H., Eaton J., Kamath P.S. (2019). Burden of liver diseases in the world. J. Hepatol..

[2] Friedman S. (2003). Liver fibrosis—From bench to bedside. J. Hepatol..

[3] Matteoni C.A., Younossi Z.M., Gramlich T., Boparai N., Liu Y.C., McCullough A.J. (1999). Nonalcoholic fatty liver disease: A spectrum of clinical and pathological severity. Gastroenterology.

[4] Younossi Z.M., Koenig A.B., Abdelatif D., Fazel Y., Henry L., Wymer M. (1999). Global epidemiology of nonalcoholic fatty liver disease-meta-analytic assessment of prevalence, incidence, and outcomes. Hepatology.

[5] Younossi Z.M. (2019). Nonalcoholic fatty liver disease-a global public health perspective. J. Hepatol..

[6] Hagström H., Nasr P., Ekstedt M., Hammar U., Stål P., Hultcrantz R., Kechagias S. (2017). Fibrosis stage but not nash predicts mortality and time to development of severe liver disease in biopsy-proven nafld. J. Hepatol..

[7] Patel S., Jinjuvadia R., Patel R., Liangpunsakul S. (2016). Insulin resistance is associated with significant liver fibrosis in chronic hepatitis c patients: A systemic review and meta-analysis. J. Clin. Gastroenterol..

[8] Carr R.M., Correnti J.C. (2015). Insulin resistance in clinical and experimental alcoholic liver disease. Ann. N. Y. Acad. Sci..

[9] Takemoto S., Yamamoto A., Tomonaga S., Funaba M., Matsui T. (2013). Magnesium deficiency induces the emergence of mast cells in the liver of rats. J. Nutr. Sci. Vitaminol..

[10] Shigematsu M., Tomonaga S., Shimokawa F., Murakami M., Imamura T., Matsui T., Funaba M. (2018). Regulatory responses of hepatocytes, macrophages and vascular endothelial cells to magnesium deficiency. J. Nutr. Biochem..

[11] Turecky L., Kupcova V., Szantova M., Uhlikova E., Viktorinova A., Czirfusz A. (2006). Serum magnesium levels in patients with alcoholic and non-alcoholic fatty liver. Bratisl. Lek. Listy.

[12] Eshraghian A., Nikeghbalian S., Geramizadeh B., Malek-Hosseini S.A. (2018). Serum magnesium concentration is independently associated with non-alcoholic fatty liver and non-alcoholic steatohepatitis. United Eur. Gastroenterol. J..

[13] Liu M., Yang H., Mao Y. (2019). Magnesium and liver disease. Ann. Transl. Med..

[14] Li W., Zhu X., Song Y., Fan L., Wu L., Kabagambe E.K., Hou L., Shrubsole M.J., Liu J., Dai Q. (2018). Intakes of magnesium, calcium and risk of fatty liver disease and prediabetes. Public Health Nutr..

[15] Wu L., Zhu X., Fan L., Kabagambe E.K., Song Y., Tao M., Zhong X., Hou L., Shrubsole M.J., Liu J. (2017). Magnesium intake and mortality due to liver diseases: Results from the third national health and nutrition examination survey cohort. Sci. Rep..

[16] Nielsen F.H. (2018). Magnesium deficiency and increased inflammation: Current perspectives. J. Inflamm. Res..

[17] Kim H.N., Kim S.H., Eun Y.M., Song S.W. (2018). Effects of zinc, magnesium, and chromium supplementation on cardiometabolic risk in adults with metabolic syndrome: A double-blind, placebo-controlled randomised trial. J. Trace Elem. Med. Biol..

[18] Nielsen F.H., Johnson L.K., Zeng H. (2010). Magnesium supplementation improves indicators of low magnesium status and inflammatory stress in adults older than 51 years with poor quality sleep. Magnes. Res..

[19] Mooren F.C., Krüger K., Völker K., Golf S.W., Wadepuhl M., Kraus A. (2011). Oral magnesium supplementation reduces insulin resistance in non-diabetic subjects - a double-blind, placebo-controlled, randomized trial. Diabetes Obes. Metab..

[20] Veronese N., Watutantrige-Fernando S., Luchini C., Solmi M., Sartore G., Sergi G., Manzato E., Barbagallo M., Maggi S., Stubbs B. (2016). Effect of magnesium supplementation on glucose metabolism in people with or at risk of diabetes: A systematic review and meta-analysis of double-blind randomized controlled trials. Eur. J. Clin. Nutr..

[21] Song Y., He K., Levitan E.B., Manson J.E., Liu S. (2006). Effects of oral magnesium supplementation on glycaemic control in type 2 diabetes: A meta-analysis of randomized double-blind controlled trials. Diabetes Metab..

[22] Pittas A.G., Lau J., Hu F.B., Dawson-Hughes B. (2007). The role of vitamin d and calcium in type 2 diabetes. A systematic review and meta-analysis. J. Clin. Endocrinol. Metab..

[23] Kim M.J., Lee K.J. (2020). Analysis of the dietary factors associated with suspected pediatric nonalcoholic fatty liver disease and potential liver fibrosis: Korean national health and nutrition examination survey 2014–2017. BMC Pediatr..

[24] Chen T.C., Clark J., Riddles M.K., Mohadjer L.K., Fakhouri T.H.I. (2020). National health and nutrition examination survey, 2015−2018: Sample design and estimation procedures. National center for health statistics. Vital Health Stat 2.

[25] Xiao G., Zhu S., Xiao X., Yan L., Yang J., Wu G. (2017). Comparison of laboratory tests, ultrasound, or magnetic resonance elastography to detect fibrosis in patients with nonalcoholic fatty liver disease: A meta-analysis. Hepatology.

[26] Jiang W., Huan S., Teng H., Wang P., Wu M., Zhou X., Ran H. (2018). Diagnostic accuracy of point shear wave elastography and transient elastography for staging hepatic fibrosis in patients with non-alcoholic fatty liver disease: A meta-analysis. BMJ Open.

[27] Tsochatzis E.A., Gurusamy K.S., Ntaoula S., Cholongitas E., Davidson B.R., Burroughs A.K. (2011). Elastography for the diagnosis of severity of fibrosis in chronic liver disease: A meta-analysis of diagnostic accuracy. J. Hepatol..

[28] Eddowes P.J., Sasso M., Allison M., Tsochatzis E., Anstee Q.M., Sheridan D., Guha I.N., Cobbold J.F., Deeks J.J., Paradis V. (2019). Accuracy of fibroscan controlled attenuation parameter and liver stiffness measurement in assessing steatosis and fibrosis in patients with nonalcoholic fatty liver disease. Gastroenterology.

[29] Ahluwalia N., Dwyer J., Terry A., Moshfegh A., Johnson C. (2016). Update on nhanes dietary data: Focus on collection, release, analytical considerations, and uses to inform public policy. Adv. Nutr..

[30] (2018). Physical Activity Guidelines for Americans.

[31] Forouhi N.G., Wareham N.J. (2014). Epidemiology of diabetes. Medicine (Abingdon).

[32] Centers for Disease Control and Prevention (2013). Testing for hcv infection: An update of guidance for clinicians and laboratorians. MMWR.

[33] Krajden M., McNabb G., Petric M. (2005). The laboratory diagnosis of hepatitis b virus. Can. J. Infect. Dis. Med. Microbiol..

[34] Division of the National Health and Nutrition Examination Surveys (2020). The National Health and Nutrition Examination Survey (Nhanes) Analytic and Reporting Guidelines.

[35] Tao M.H., Dai Q., Millen A.E., Nie J., Edge S.B., Trevisan M., Shields P.G., Freudenheim J.L. (2015). Associations of intakes of magnesium and calcium and survival among women with breast cancer: Results from western new york exposures and breast cancer (web) study. Am. J. Cancer Res..

[36] Rodríguez-Hernández H., Gonzalez J.L., Rodríguez-Morán M., Guerrero-Romero F. (2005). Hypomagnesemia, insulin resistance, and non-alcoholic steatohepatitis in obese subjects. Arch. Med. Res..

[37] Younossi Z.M., Stepanova M., Younossi Y., Golabi P., Mishra A., Rafiq N., Henry L. (2020). Epidemiology of chronic liver disease in the USA in the past three decades. Gut.

[38] Agarwal S., Reider C., Brooks J.R., Fulgoni V.L. (2015). Comparison of prevalence of inadequate nutrient intake based on body weight status of adults in the united states: An analysis of nhanes 2001–2008. J. Am. Coll. Nutr..

[39] Liu J., Zhu X., Fulda K.G., Chen S., Tao M.H. (2019). Comparison of dietary micronutrient intakes by body weight status among mexican-american and non-hispanic black women aged 19–39 years: An analysis of nhanes 2003–2014. Nutritients.

[40] Hoenderop J.G., Bindels R.J. (2005). Epithelial ca2+ and mg2+ channels in health and disease. J. Am. Soc. Nephrol..

[41] Nielsen F.H., Milne D.B., Gallagher S., Johnson L., Hoverson B. (2007). Moderate magnesium deprivation results in calcium retention and altered potassium and phosphorus excretion by postmenopausal women. Magnes. Res..

[42] Sigrist R.M.S., Liau J., Kaffas A.E., Chammas M.C., Willmann J.K. (2017). Ultrasound elastography: Review of techniques and clinical applications. Theranostics.

[43] Guenther P.M., Bowman S.A., Goldman J.D. Alcoholic Beverage Consumption by Adults 21 Years and Over in the United States: Results from the National Health and Nutrition Examinationsurvey, 2003–2006. Technical Report. Center for Nutrition Policy and Promotion and Agriculturalresearch Service, U.S. Department of Agriculture. www.Cnpp.Usda.Gov/publications/dietaryguidelines/2010/meeting5/alcoholicbeveragesconsumption.Pdf.

[44] Murakami K., Livingstone M.B. (2015). Prevalence and characteristics of misreporting of energy intake in us adults: Nhanes 2003–2012. Br. J. Nutr..

[45] Roark R.A., Niederhauser V.P. (2013). Fruit and vegetable intake: Issues with definition and measurement. Public Health Nutr..

